# Case of Traumatic Tetanus Successfully Treated by Large Quantities of Wine and Brandy, with Other Means

**Published:** 1845-10

**Authors:** James William Ilott

**Affiliations:** Bromley, Kent.


### SELECTIONS

FROM

### AMERICAN AND FOREIGN JOURNALS.

---

*Case of Traumatic Tetanus successfully treated by large quantities of Wine and Brandy, with other means.* By James William Ilott, Esq., of Bromley, Kent. Communicated by James Arthur Wilson, M.D., Physician to St. George's Hospital.

The patient, a robust man, aged 30, was thrown from his chaise, Nov. 28, and met with a compound fracture of the tibia and fibula, with considerable injury to the soft parts. By extension, the bones were placed in a good position, and it was determined to save the limb. The patient went on remarkably well until the 12th of December, when tetanic spasms first commenced. Healthy granulations had formed, and were covering the denuded bone. The spasmodic action affected the muscles for a time, and then gave way, the muscles of the jaw being those chiefly affected. On the 14th he complained of cramp and spasm in the chest. A blister was applied, and calomel and cathartic medicine administered.—15th. The pain and spasm of the chest were somewhat relieved, but he was very weak. He was ordered brandy-and-water and medicine with ether, every two or three hours, after which he somewhat rallied. He continued much the same to the 18th, when the tetanic symptoms were much increased in severity, and in the evening there was opisthotonos. Mr. John Scott saw him, agreed in the propriety of continuing the stimulants, and suggested opiate enemata, and turpentine and castor oil internally. This treatment was pursued, with little variation, till the 21st, when he was observed to be extremely low, with a pulse about forty, and in a cold clammy perspiration. The quantity of brandy at this time administered amounted to rather more than three pints in the twenty-four hours; every part of the muscular system seemed affected with spasm.—Dec. 22nd. He was much the same, but the wound was looking more healthy. Dr. Wilson saw him, and urged the continuance of the stimulants, but recommended the entire discontinuance of opiates. He also recommended a suppository, composed of quinine, sesquicarbonate of ammonia, and camphor, to be administered every three or four hours. From this period he continued to improve, and on the 26th all tetanic symptoms had disappeared. During the eight days in which the disease was at its height, the patient took two gallons of brandy, besides wine, beef-tea, jelly, &c. Jan. 29th. The wound was nearly healed, and he was daily gaining strength. In a supplement to this communication, Dr. Wilson remarks that the case affords a well-marked instance of traumatic fever, and that the principle on which it was treated was one of unremitting stimulus and support, opium and all other narcotics being entirely laid aside on the tenth day of the attack. The extent and severity of the constitutional disorder were strongly indicated by the indifference of the patient to all the ordinary effects of the alcoholic stimulus. From this case he draws the important pathological inference, that tetanus, like fever, is a disorder of the entire system. Determined especially to the muscles, it is continually operative in the other structures, as in the universal blood. Generated after a definite period of incubation, it requires time for its development—time for its cure. On these views and indications, that treatment which assumes to be rational must address itself to the general business of nutrition rather than to the partial agencies of the nerves. Under the great systematic error of our modern pathology, tetanus is encountered almost exclusively by opium and other narcotic poisons. There is no plausible theory to account for the preference of these fatal drugs. By experience they have long been utterly condemned.—[*London Lancet.*

